# Current Landscape and Evolving Role of Targeted Agents in Urothelial Carcinoma

**DOI:** 10.3390/cancers18030409

**Published:** 2026-01-28

**Authors:** Enrico Sammarco, Antonio Pellino, Eleonora Bona, Azzurra Farnesi, Elisa Biasco, Chiara Caparello, Andrea Marini, Irene Stasi, Gianna Musettini, Cecilia Barbara, Ermelinda De Maio, Luigi Coltelli, Samanta Cupini, Giacomo Allegrini

**Affiliations:** 1 Department of Oncology, Azienda USL Toscana Nord Ovest, 56121 Pisa, Italyandrea.marini@uslnordovest.toscana.it (A.M.);; 2 Medical Oncology Unit, Livorno Hospital, Azienda USL Toscana Nord Ovest, 57124 Livorno, Italy; 3 Medical Oncology Unit, Pontedera Hospital, Azienda USL Toscana Nord Ovest, 56025 Pontedera, Italy

**Keywords:** urothelial carcinoma (UC), targeted treatments, biomarkers, FGFR, HER2, Nectin-4

## Abstract

Urothelial carcinoma represents one of the deadliest urological tumors, due to its biological aggressiveness and the ephemeral response to platinum-based chemotherapy of advanced-stage disease. The use of immune checkpoint inhibitors has positively impacted the prognosis of these patients, although only a proportion of them achieve long lasting disease control. As with other solid tumors, in the last decade, there has been a growing interest in the molecular characterization of urothelial carcinoma in order to identify new therapeutic targets and improve survival in these patients. The aim of this narrative review is to summarize the main evidence available in the literature concerning the role of targeted therapies in urothelial carcinoma, especially focusing on related molecular targets, published data on clinical efficacy, and on future perspectives. Specifically, the effort of this review aims to strengthen the clinical impact of well-known molecular patterns and relative target treatments (such as FGFR inhibitors and Nectin-4-directed antibody–drug conjugates) and to examine the role and preliminary data of drugs targeting biomarkers that are not yet commonly used in clinical practice (such as HER2).

## Simple Summary

Despite the availability of various treatments, such as chemotherapy and immunotherapy, urothelial carcinoma remains a disease with a poor prognosis. In recent years, greater biological knowledge has led to the identification of new therapeutic targets, in order to improve survival of these patients by personalizing their systemic treatment. In this review, we aim to collect the main available data about the efficacy and safety of target treatments in urothelial carcinoma, also focusing on their possible future applications.

## 1. Introduction

Urothelial carcinoma (UC) represents the most common histological type of cancer arising from the transitional epithelium lining the urinary tract; in most cases, UC originates from the bladder, while in 5–10%, it can be derived from the neoplastic transformation of the transitional cells of the upper urinary tract (renal pelvis, renal calyces, and ureter). Bladder UC is the 10th-most common cancer worldwide [1], accounting for about 570,000 new cases and 210,000 deaths each year; upper urinary tract urothelial carcinoma (UTUC) is quite an uncommon tumor [2] with an estimated annual incidence of almost two cases per 100,000 inhabitants in Western countries.

When non-muscle-invasive bladder cancer (NMIBC) occurs, the optimal management is the complete excision of all intravesical lesions by transurethral resection of the bladder tumor (TURB), followed by intravesical instillations of chemotherapy or bacillus Calmett-Guerin (BCG) when indicated. Radical cystectomy (RC) [3] is required in case of very high-risk NMIBC o in BCG-unresponsive patients. For this group of patients with BCG-unresponsive high-risk NMIBC who were ineligible or declined to undergo RC, pembrolizumab monotherapy [4,5] has showed an interesting antitumor activity.

RC is the standard of care in patients with muscle-invasive bladder cancer (MIBC); cisplatin-based neoadjuvant chemotherapy is recommended for patients who are considered fit for cisplatin. The role of adjuvant chemotherapy [6,7] in patients who have not received neoadjuvant therapy is debated due to the lower quality of supporting evidence. Adjuvant immunotherapy with nivolumab [8,9] improved disease-free survival (DFS) and overall survival (OS) in patients ineligible for (or who have declined) cisplatin-based adjuvant therapy or in patients with residual muscle-invasive disease (and/or node positive disease) after neoadjuvant chemotherapy. Perioperative immunotherapy with durvalumab in combination with neoadjuvant cisplatin-gemcitabine [10] prolonged event-free survival (EFS) compared to neoadjuvant chemotherapy alone.

Radical nephroureterectomy (RNU) [11] is recommended in patients with high-risk UTUC; platinum- and gemcitabine-based adjuvant chemotherapy [12] showed a significant improvement in DFS in patients with locally advanced UTUC after RNU.

Advanced or metastatic UC remains a highly aggressive disease, characterized by a poor prognosis; first-line platinum-based chemotherapy has remained the standard of care for decades, followed by single agent immunotherapy [7] (both as maintenance therapy in case of disease control and as a second-line treatment in case of rapid disease progression). Enfortumab Vedotin, a nectin-4-directed antibody–drug conjugate (ADC), represents third-line treatment [7,13] after platinum-based chemotherapy and immunotherapy. More recently, pembrolizumab plus enfortumab vedotin [14] outperformed platinum-based chemotherapy as a first-line treatment in advanced UC.

In recent years, there has been a growing interest in the molecular characterization of UC in order to identify new therapeutic targets. In this review, we aim to offer an overview of the emerging molecular target therapies in UC, focusing on the data already available in the literature and on the most interesting future perspectives capable of changing the therapeutic landscape of this disease.

## 2. Literature Search and Selection of Trials

All published data about the use of targeted treatment in UC (full text of articles and abstracts presented at international conferences) were collected and summarized using PubMed as database. Additionally, the main ongoing clinical trials were selected by using the electronic database of clinical trials.

## 3. Fibroblast Growth Factor Receptor (FGFR) Inhibition

The FGFR signaling pathway represents one of the most interesting molecular targets in the therapeutic landscape of UC. Up to 20% of advanced urothelial bladder carcinoma have genetic alterations of FGFR3 (especially activating missense mutations in exons 7, 10, and 15) [15] with higher incidence in high-grade UTUC [16] (about 35%). Base substitution is the most common alteration [17], in particular, the replacement of serine with cysteine in codon 249 (S249C).

The clinical activity and safety of several FGFR inhibitors has been evaluated over the last few years in patients with diagnosis of UC.

### 3.1. FGFR Inhibition in Advanced UC

The activity and safety of erdafitinib, an oral inhibitor of various FGFR isoforms 1–4 [18], was preliminary described in a multicenter, four-part phase I clinical trial that included 187 patients with pretreated advanced solid tumors. In part 2 and 3, a daily regimen of 9 mg was administered to patients with KRAS wild-type tumors harboring FGFR alterations (amplifications, activating mutations, gene fusions, or other molecular alterations leading to activation of the FGFR pathway). In part 4 (enrolling only patients with FGFR-activating mutations or fusions), an intermittent schedule (7 days on/7 days off) of 10 mg erdafitinib was used. The overall response rate (ORR) was 46.2% (12/26) in the subgroup of UC harboring FGFR mutations or fusions [19], with a median duration of response of 5.6 months. Globally, hyperphosphatemia (64%), dry mouth (42%) and stomatitis (37%) were the most common treatment-emergent adverse events (TEAEs).

In the open-label, phase II BLC2001 study, 101 patients with advanced or metastatic UC with FGFR3 mutation or FGFR2/3 fusion were treated with a starting dose of 8 mg per day of a continuous administration of oral erdafitinib [20] that could be escalated to 9 mg (if phosphate level was less than 5.5 mg per deciliter). Most patients had been treated with one (48%) or more (43%) lines of previous systemic therapy, while 10% of patients were naïve to systemic treatment; in 24 out of 101 patients, a progression of disease recurred during or after immunotherapy. The primary endpoint, the ORR observed in the total population, was 40% (4% of complete response and 36% of partial response), with a median duration of response (DoR) of 6 months; median PFS was 5.5 months (95% CI, 4.2 to 6.0) and median OS was 13.8 months (95% CI, 9.8 to not reached). The most common grade 3–4 adverse events were stomatitis (14%), hyponatremia (11%), and asthenia (8%), while 78% of patients developed any grade of hyperphosphatemia (only two grade 3 events occurred). Discontinuation of treatment due to adverse events [21] (detachment of the retinal pigment epithelium, hand-foot syndrome, dry mouth, and skin or nail toxicity) was observed in 13 patients.

In the phase III THOR trial [22,23], for the first time, the efficacy and safety of an FGFR inhibitor was evaluated, in comparison to the standard of care of the treatment, in a large group of patients with pretreated advanced UC expressing selected FGFR2/3 alterations (mutations or fusions). In cohort 1, including 266 patients who previously received immune checkpoint inhibitor (ICI) and chemotherapy (in combination or in a maintenance setting, while immunotherapy alone was only allowed for subjects with documented cisplatin ineligibility), erdafitinib (at same dosing of BLC2001) improved OS (median OS 12.1 vs. 7.8 months, HR 0.64 95% CI 0.47–0.88 *p* = 0.005), PFS (median PFS 5.7 vs. 2.7 months, HR 0.58 95% CI 0.44–0.78 *p* = 0.0002), and ORR (45.6% vs. 11.5%) compared to the investigator’s choice of chemotherapy (docetaxel or vinflunine). Overall survival favored erdafitinib across all subgroups (regardless of the kind of FGFR alteration, number of previous lines, and visceral disease); furthermore, a clear advantage was observed in primary UTUC patients (median OS 23.3 vs. 7.2 months, HR 0.34 95% CI 0.18–0.64). Discontinuation rate due to TRAEs was 8.1% (11/135); palmar-plantar erythrodysesthesia (9.6%), stomatitis (8.1%), onycholysis (5.9%), and hyperphosphatemia (5.2%) were the most frequent grade 3–4 adverse events. Nail and skin alterations (11%) were reported as the most common grade 3–4 adverse events of special interest, while three patients reported a severe central serous retinopathy. Crossover to erdafitinib arm was allowed, following these promising data. In cohort 2 of THOR [24] (351 patients with ICI-naïve advanced UC previously treated with one line of chemotherapy), erdafinib was not superior to pembrolizumab in terms of survival (median OS 10.9 vs. 11.1 months, HR 1.18 95% CI 0.92–1.51 *p* = 0.18); erdafitinib was associated with a numerically longer PFS (median PFS 4.4 vs. 2.7 months, HR 0.88 95% CI 0.70–1.10), higher ORR (40.0% vs. 21.6%), and shortened duration of response (median value 4.3 vs. 14.4 months) compared to pembrolizumab. No new safety signals were observed for both treatments.

The safety and activity of Rogaratinib, another oral pan-FGFR inhibitor, were initially evaluated in a phase I dose escalation and dose expansion trial. In the UC cohort [25], 52 patients with a previously treated tumor harboring FGFR1–3 mRNA overexpression or an activating point mutation in FGFR3 were treated with rogaratinib at the RP2D (800 mg twice in continuous 3-week cycles), with an ORR in UC patients of 24%. The most common grade 3 or worse TEAEs were fatigue (10%) and asymptomatic increased lipase (8%); any grade of hyperphosphatemia was observed in 77 patients (61%), with only one grade 3 event. Subsequently, in FORT-1, a phase II/III, randomized, open-label trial, patients with platinum-pretreated metastatic UC and FGFR1/3 mRNA expression were assigned to receive rogaratinib or chemotherapy (taxanes or vinflunine); almost half of the patients also received ICI-based therapy. Because of the similar efficacy between the two treatments (ORR 20.7% vs. 19.7%; median OS 8.3 vs. 9.8 months) [26], enrollment was stopped prior to progression to the phase III part.

The phase II FIGHT-201 evaluated the antitumor activity of pemigatinib in patients with metastatic UC who previously received at least one prior line of systemic therapy (or were ineligible to receive platinum-based chemotherapy) [27], whose tumor harbored FGFR3 mutations or fusions/rearrangements (cohort A) or other FGFR alterations (cohort B). Overall, 204 patients were enrolled in cohort A; the ORR was 17.8% and 23.3% in patients who received continuous and intermittent administration of pemigatinib. The most common grade 3 or higher TEAEs were stomatitis (8.8%), urinary tract infection (7.3%), fatigue (5.0%), and diarrhea (3.8%).

Activity of infigratinib, a potent FGFR1-3 inhibitor, in UC was reported from the expansion cohort [28] of a phase Ib clinical trial. The ORR in 67 patients with advanced UC harboring activating FGFR3 mutation/fusion was 25.4%. The most common grade 3/4 TEAEs were hyperlipasemia (10.4%), anemia (7.5%), and hyperphosphatemia (7.5%).

Preclinical data suggest an important role of the FGFR signaling pathway in modifying the tumor microenvironment, through the induction of immunogenic cell death and the consequent release of damage-associated molecular patterns (DAMPs) capable of enhancing the adaptive immune response. Therefore, the use of FGFR inhibitors [29] could increase sensitivity and overcome the resistance to ICIs. In the phase II Norse trial, 87 cisplatin-ineligible patients with FGFR-altered metastatic UC were randomized to receive erdafitinib monotherapy or erdafitinib plus cetrelimab (an anti PD-1 monoclonal antibody). The ORR and 1-year OS rate were slightly higher for the combination treatment over erdafitinib monotherapy [30] (ORR 54.5 vs. 44.2%; 1-year OS rate 68% vs. 56%).

A phase Ib trial will establish the feasibility and safety of erdafitinib in combination with enfortumab vedotin in metastatic UC, with FGFR2/3 activating genomic alterations whose disease progressed after platinum-based chemotherapy and immunotherapy (ClinicalTrials.gov identifier NCT04963153).

### 3.2. FGFR Inhibition in Perioperative/Adjuvant Treatment

Erdafitinib efficacy in the earlier stage of UC was reported in interim analysis of phase II THOR-2, showing interesting activity in FGFR3/2-altered intermediate risk (exploratory cohort 3) [31] and high-risk (exploratory cohort 2) [32] NMIBC patients. Recently, erdafitinib prolonged recurrence-free survival (median RFS not reached vs. 11.6 months, HR 0.28 95% CI 0.1–0.6 *p* = 0.0008) compared to investigator’s choice intravesical chemotherapy (gemcitabine or mitomycin c) in cohort 1 of this trial [33], that enrolled 73 patients with BCG-unresponsive NMIBC who refuse or are ineligible for RC. However, slow accrual led to early closure of THOR-2.

In an open label, phase I trial, intravesical delivery of erdafitinib with TAR-210 was well tolerated and was associated with a high rate of complete response (over 80%) [34] in patients with recurrent intermediate or high-risk NMIBC with selected FGFR alterations.

Role of adjuvant treatment with infigratinib in patients with high-risk muscle-invasive UTUC or urothelial bladder cancer after radical surgery carrying susceptible FGFR3 alterations (activating mutations, gene fusions or rearrangements) was explored in phase III PROOF 302 trial [35,36]; enrollment was stopped due to insufficient accrual.

The antitumor activity of erdafitinib with or without cetrelimab as neoadjuvant treatment in patients with MIBC harboring with susceptible FGFR3/2 alterations, who are ineligible for (or decline) cisplatin-based neoadjuvant chemotherapy, is being studied in the phase II SOSUG-NEOWIN trial; the coprimary endpoint are the pathologic complete response (pCR) and tumor downstaging rate after radical cystectomy (EU Clinical Trials register 2022-002586-15).

Erdaftinib received approval in Europe and North America in patients with advanced UC harboring susceptible FGFR3 genetic alterations who were previously treated with at least one line of systemic treatment, including an ICI; this drug represents the first biomarker-driven therapy to be authorized in metastatic UC. Ongoing trials will clarify the role of erdafitinib (as monotherapy or in combination with other agents, as ICIs) in the earlier setting. Differences in terms of pharmacokinetics and pharmacodynamics [37] could explain the different results in efficacy and safety between the various inhibitors. Furthermore, different testing methods to identify FGFR alterations were used across various trials (for rogaratinib trials, patient selection was based on the overexpression of FGFR mRNA; for erdafitinib trials, fusions or mutations were found by using RNA sequencing). Additionally, previous exposure to ICI, as required in cohort 1 of THOR trial, could identify patients likely to benefit the most from FGFR inhibitors.

## 4. Nectin-4 Targeting

The role of Nectin cell adhesion molecule-4 (Nectin-4) overexpression in tumor proliferation and metastatic dissemination is recognized in several tumor types [38], including UC, breast, lung, and colorectal cancer.

Enfortumab vedotin is a novel ADC that targets Nectin-4 on tumor cells and induces apoptosis by delivering a microtubule-disrupting agent, monomethyl auristatin E (MMAE). In a phase I study [39], intravenous administration of enfortumab vedotin at 1.25 mg/kg on days 1, 8, and 15 of the 28-day cycle (recommended phase 2 dose, RP2D) showed a confirmed ORR of 43% and a median OS of 12.3 months in 112 patients with metastatic UC previously treated with ICIs (cohort C). Fatigue (53%), alopecia (46%), decreased appetite (42%), dysgeusia (38%), nausea (38%), and peripheral sensory neuropathy (38%) were the most common TRAEs; hyperglycemia (5%) was the only grade 3–4 TRAE that occurred in almost 5% of patients, whereas peripheral sensory neuropathy was the most common TRAE leading to discontinuation. The presence of high-level expression of Nectin-4 in tumor samples, initially required, was no longer necessary after a subsequent amendment, because of high Nectin-4 expression in most UC tumor samples.

In phase II EV-201 [40], 125 patients with advanced UC previously treated with platinum-based chemotherapy and ICI (cohort 1) received enfortumab vedotin, with a confirmed ORR of 44%. Among grade ≥ 3 TRAEs, the most common were fatigue (6%), maculopapular rash (4%), and peripheral sensory neuropathy (2%). Results from cohort 2 [41] (enrolling 89 cisplatin-ineligible patients who had not received platinum-based treatment and whose advanced UC was previously treated with immunotherapy) confirmed interesting activity of enfortumab vedotin (ORR 52%). The phase III EV-301 [13] randomized 608 patients with advanced UC previously treated with platinum-based therapy and disease progression during or after ICI to receive enfortumab vedotin or investigator-chosen chemotherapy (docetaxel, paclitaxel, or vinflunine). Enfortumab vedotin showed significant improvement in OS, primary endpoint, compared to standard chemotherapy (median OS 12.88 vs. 8.97 months, HR 0.70 95% CI 0.56–0.89 *p* = 0.001); PFS was also longer in the enfortumab vedotin arm (median PFS 5.55 vs. 3.71 months, HR 0.62 95% CI 0.51–0.75 *p* < 0.001). The incidence of all grade (93.9% vs. 91.8%) and grade ≥ 3 (51.4% vs. 49.8%) TRAEs was similar between the two treatment arms; maculopapular rash was the most common grade ≥ 3 TRAE (7.4%) in the enfortumab vedotin group, then fatigue (6.4%), decreased neutrophil count (6.1%), and peripheral sensory neuropathy (3%). Due to the high expression of Nectin-4 on the skin, cutaneous adverse events are considered a typical class toxicity [42] of ADCs directed against Nectin-4; peripheral neuropathy is related to microtubule-disrupting action of MMAE, a payload of enfortumab vedotin. Treatment-related hyperglycemia occurred in 6.4% of patients who received enfortumab vedotin. After a median follow up of 2 years [43], enfortumab vedotin treatment has maintained a clinical meaningful improvement in OS and PFS compared to standard chemotherapy, with higher ORR (41% vs. 18%) and quite similar duration of response.

Immunogenic cell death and antibody-dependent cell-mediated cytotoxicity induced by ADCs may improve the antitumor activity of immunotherapy [44], increasing the infiltration of T cells into the tumor microenvironment. In the dose expansion part of EV-103 [45], enfortumab vedotin in combination with pembrolizumab showed promising activity (ORR 73.3%) and clinical efficacy (median PFS 12.3 months, median OS 26.1 months) in 45 cisplatin-ineligible patients with previously untreated metastatic UC; the most common TRAEs were peripheral sensory neuropathy (55.6%), fatigue (51.1%), and alopecia (48.9%). In cohort K of EV-103 [46], 149 cisplatin-ineligible patients were randomly assigned to receive enfortumab vedotin in combination with pembrolizumab or as monotherapy in first-line treatment. The confirmed ORR was 64.5% and 45.2% for combination treatment and enfortumab vedotin monotherapy, respectively; in the pembrolizumab plus enfortumab arm, the most common grade 3 or higher TRAEs were maculopapular rash (17.1%), fatigue (9.2%), and neutropenia (9.2%).

In a large global, open label, phase III trial (EV-302) [14,47], 886 patients with previously untreated locally advanced or metastatic UC were randomized to receive pembrolizumab plus enfortumab vedotin or chemotherapy with cisplatin or carboplatin plus gemcitabine; the dual primary endpoints were PFS and OS. Pembrolizumab plus enfortumab vedotin significantly prolonged OS (median OS 31.5 vs. 16.1 months, HR 0.47 95% CI 0.38–0.58 *p* < 0.00001) and PFS (median PFS 12.5 vs. 6.3 months, HR 0.45 95% CI 0.38–0.54 *p* < 0.00001) compared to standard chemotherapy; additionally, confirmed ORR was higher (67.7% vs. 44.4%) with the novel combination therapy, with a longer duration of response (23 vs. 7 months). The incidence of grade 3 or higher TRAEs was lower with pembrolizumab plus enfortumab (55.9% vs. 69.5%); the most common TRAEs were maculopapular rash (7.7%), hyperglycemia (5.0%), and neutropenia (4.8%). Among grade 3 or higher TRAEs of special interest for enfortumab vedotin, skin reactions (15.5%), peripheral neuropathy (6.8%), and hyperglycemia (6.1%) were reported. This combination continued to show improved OS, PFS, and ORR [48] over platinum-based chemotherapy after a median follow up of 2.5 years.

Zelenectide pevedotin, another Nectin-4 targeting agent conjugated with MMAE, demonstrated promising antitumor activity in combination with pembrolizumab in the expansion cohort [49] of the ongoing phase I–II Duravelo-1. Zelenectide pevedotin is currently under evaluation in phase II–III Duravelo-2, as a first-line treatment in combination with pembrolizumab in platinum-eligible patients (cohort 1) or as monotherapy in previously treated patients (cohort 2) (ClinicalTrials.gov identifier NCT06225596).

Neoadjuvant treatment with enfortumab vedotin [50] showed preliminary data of activity in 22 cisplatin-ineligible patients with MIBC in cohort H of EV-103; the pCR rate, primary endpoint, was 36.4%. Recently, in phase III EV-303 [51], perioperative pembrolizumab plus enfortumab vedotin significantly improved EFS (median EFS NR vs. 15.7 months, HR 0.40 95% CI 0.28–0.57 *p* < 0.001) compared to radical cystectomy alone in 344 patients with MIBC who were cisplatin-ineligible (or declined cisplatin); additionally, perioperative treatment resulted in longer OS (median OS NR vs. 41.7 months, HR 0.50 95% CI 0.33–0.74 *p* < 0.001) and higher pCR rate (57.1% vs. 8.6%; estimated difference 48.3%; 95% CI 39.5–56.5 *p* < 0.001), with manageable safety profile (incidence of grade 3 or higher TRAEs was 71.3% and 45.9%, respectively). Allocation to a third trial arm with perioperative pembrolizumab monotherapy was stopped in 2022.

Based on these results, the ongoing open-label, phase III EV-304 is planned to randomize about 780 cisplatin-eligible patients with MIBC to receive perioperative treatment with pembrolizumab plus enfortumab or standard chemotherapy with cisplatin plus gemcitabine. Primary endpoints of this trial are pCR and event-free survival (EFS), whereas secondary endpoints are overall survival, disease-free survival, pathological downstaging, and safety (ClinicalTrials.gov identifier NCT04700124).

Enfortumab vedotin is currently approved in combination with pembrolizumab as a first-line treatment in advanced disease and as monotherapy in patients with metastatic UC who previously received platinum-based chemotherapy and immunotherapy. In future, the results from ongoing trials could also extend the indication of pembrolizumab-enfortumab vedotin combination in the perioperative setting.

## 5. TROP-2 Targeting

The high expression of surface protein TROP-2 in several cancer types led to the development of drugs binding selectively to this target; more than 80% of tumor samples [52] from patients with advanced UC overexpress TROP-2.

Sacituzumab govitecan, a novel ADC directed against TROP-2, uses SN-38, the active metabolite of the topoisomerase 1 inhibitor irinotecan, as cytotoxic payload. In cohort 1 of TROPHY-U-01 [53], sacituzumab govitecan showed promising activity in 113 patients with advanced UC who had progressed after prior platinum-based chemotherapy and ICI, with ORR of 27% and median PFS and OS of 5.4 and 10.9 months, respectively. The most common grade 3 or higher TRAEs were neutropenia (35%), leukopenia (18%), anemia (14%), diarrhea (10%), and febrile neutropenia (10%). Safety profile (hematological and gastrointestinal adverse events) is mainly attributable to toxicity of SN-38 [42], rather than to a class effect linked to TROP-2 targeting. Additionally, sacituzumab govitecan was associated with interesting results in activity and efficacy among 38 platinum-ineligible patients whose disease progressed after ICI in cohort 2 [54] (ORR 32%, median PFS 5.6 months and median OS 13.5 months).

In the phase III TROPiCS-04 [55], a large, global, open-label phase III trial, 711 patients with advanced UC previously treated with platinum-based chemotherapy and immunotherapy were randomized to receive sacituzumab govitecan or investigator’s choice chemotherapy (docetaxel, paclitaxel, or vinflunine); the primary endpoint, OS, was not met (median OS 10.3 vs. 9.0 months, HR 0.86 95% CI 0.73–1.02 *p* = 0.087). Sacituzumab govitecan was associated with higher incidence of grade ≥ 3 TRAEs (67% versus 35%), especially neutropenia (35%), diarrhea (15%), anemia (13%), and febrile neutropenia (12%).

Preliminary data about combining sacituzumab govitecan with other drugs were recently presented. In cohort 3 of TROPHY-U-01, pembrolizumab plus sacituzumab govitecan demonstrated encouraging results in 41 ICI-naïve patients with platinum-pretreated advanced UC in cohort 3 [56] (ORR 41%, median PFS 5.3 months and median OS 12.7 months); the most common grade ≥ 3 TRAEs were neutropenia (37%; 10% febrile neutropenia), leukopenia (20%), and diarrhea (20%). Interim analysis of phase II JAVELIN Bladder Medley [57] showed interesting results in terms of PFS (median value 11.17 months) with maintenance treatment with avelumab plus sacituzumab govitecan in 74 patients with advanced UC without progression following first-line platinum-based chemotherapy.

In a phase I trial [58], a double ADC combination with sacituzumab govitecan and enfortumab vedotin showed a manageable safety profile and promising activity in 23 patients with pretreated metastatic UC.

The first results of ongoing phase II SURE-01 [59] demonstrated interesting activity of neoadjuvant treatment with sacituzumab govitecan in muscle-invasive bladder cancer, resulting in a pCR of 36% in 33 patients who were considered ineligible for (or refused) cisplatin-based chemotherapy.

Sacituzumab govitecan is currently under evaluation as a neoadjuvant treatment in combination with perioperative pembrolizumab in patients who were considered ineligible for (or refused) cisplatin-based chemotherapy in phase II SURE-02 (ClinicalTrials.gov identifier NCT05535218), as a first-line treatment in combination with nivolumab plus ipilimumab (ClinicalTrials.gov identifier NCT04863885) or with novel ICIs (anti PD-1 zimberelimab, anti TIGIT domvanalimab) for cisplatin-ineligible patients (cohort 6 of TROPHY-U-01). Furthermore, sacituzumab govitecan is being assessed as maintenance treatment in combination with avelumab or novel anti PD-1 zimberlimab in cohort 5 of TROPHY-U-01.

After initial FDA-accelerated approval, negative data of TROPiCS-04 led to the withdrawal of sacituzumab govitecan authorization in the USA in previously treated metastatic UC; preliminary data of sacituzumab govitecan combinations need to be confirmed in large randomized trials to clarify the potential role of this drug in UC. Based on currently available data, the use of ADCs directed against TROP-2 seems to be associated with lower efficacy than those against Nectin-4, reflecting different activity and a different safety profile with very different class toxicities.

## 6. HER2 Inhibition

The expression of the human epidermal growth factor receptor 2 (HER2) gene is a well-known therapeutic target in several solid tumors; the incidence of its overexpression, detected by immunochemistry (IHC) or fluorescent in situ hybridization (FISH), in UC is very variable (up to 38%) [60] and is higher in patients with more aggressive disease (MIBC or metastatic UC) and in UTUC.

The efficacy and safety of the addition of trastuzumab (a humanized monoclonal antibody against the HER2 receptor) to platinum plus gemcitabine first-line chemotherapy [61] were evaluated in a randomized phase II study. Out of 563 screened patients, 75 (13%) met the main inclusion criterion (HER2 overexpression on IHC of primary tumor confirmed by FISH). The major limitation of this trial was the low rate of HER2 overexpressing tumor detected (13%); no significant differences in PFS (primary endpoint) were observed between triplet and doublet therapies (median PFS 8.2 vs. 10.2 months, *p* = 0.689). Previously, in a single-arm phase II study, trastuzumab in association with carboplatin and paclitaxel [62] had shown promising results as first-line treatment in 57 advanced HER2-positive UC patients in terms of ORR (70%), PFS (median value 9.3 months), and OS (median value 14.1 months), with a favorable safety profile. More recently, a trastuzumab biosimilar in combination with paclitaxel [63] was associated with an ORR of 48.1% in 27 patients with platinum-pretreated metastatic HER2-positive UC.

Despite negative results from phase II studies [64], lapatinib (a dual tyrosine kinase inhibitor which blocks the HER2 and epidermal growth factor receptor pathways) was chosen as maintenance therapy in a randomized, placebo-controlled phase III trial [65] that included 232 patients with HER1/2-positive advanced UC without radiologic progression of disease after four to eight cycles of first-line chemotherapy. Addition of lapatinib did not produce significant improvement in PFS (primary endpoint; median PFS 4.5 vs. 5.1 months, HR 1.07 95% CI 0.81–1.43 *p* = 0.63) or OS (median OS 12.6 vs. 12 months, HR 0.96 95% CI 0.70–1.31 *p* = 0.80) compared to placebo.

Dual HER2 blockade with pertuzumab plus trastuzumab [66] showed clinical activity (ORR 18%) in the cohort of 22 patients with refractory HER2-positive metastatic UC in a phase IIa basket clinical trial.

In recent years, similar to other solid tumors, preliminary interesting data on the use of novel antibody–drug conjugates (ADCs) targeting HER2 have emerged in the management of advanced UC. In the single-arm phase II KAMELEON trial [67], 13 patients with pretreated HER2-positive (by IHC assay) metastatic UC received trastuzumab emtansine. The best overall response (BOR), the primary endpoint of this trial, was partial response achieved by five patients (39%) with a median OS of 7 months. Due to difficult enrollment, this trial closed its accrual prematurely. Disitamab Vedotin (RC48-ADC), a novel humanized anti-HER2 antibody conjugated with monomethyl auristatin E, demonstrated promising clinical activity in a multicenter, single-arm, phase II trial [68], which enrolled 43 patients with HER2 overexpressing metastatic UC who previously failed at least one line of chemotherapy; the primary endpoint was ORR. Disitamab Vedotin treatment was associated with an ORR of 51.2% (22/43), while median PFS and OS were 6.9 and 13.9 months, respectively. The most common TRAEs were hypoesthesia (60.5%) and alopecia (55.8%); grade 3 AEs occurred in 25 patients (including hypoesthesia, 23.3%, and neutropenia, 14.0%). A pooled analysis of two phase II trials [69] confirmed antitumor activity and a manageable safety profile of disitamab Vedotin with a similar ORR (50.5%). Trastuzumab deruxtecan therapy demonstrated a similar rate of activity (39%) in a cohort of 41 patients with HER2-expressing UC after at least one prior line of systemic treatment [70] in a phase II study (DESTINY-PanTumor02).

Combining ADCs with immunotherapy could overcome the onset of resistance mechanisms [44], exploiting a synergistic action between the two classes of drugs. Trastuzumab deruxtecan in combination with nivolumab [71] showed good clinical efficacy (ORR 36.7%, median PFS 6.9 months, median OS 11 months) in a phase Ib trial that included 30 platinum-pretreated metastatic UC patients with high expression of HER2 by IHC; the most frequent TEAEs were nausea (73.5%), fatigue (52.9%), and vomiting (44.1%), while drug-related interstitial lung disease (ILD) occurred in 23.5% of all patients (one grade 5 event). A similar combination approach, disitamab vedotin plus toripalimab [72] (anti PD-1 monoclonal antibody), was associated with a confirmed ORR of 73.2% (9.8% complete response) in 41 patients with advanced UC (61% of all patients were systemic treatment-naïve); although patients were included regardless of HER2 positivity, most responses were observed in cases with greater expression of HER2 by IHC assay. The preliminary results of cohort C of the phase II trial RC48G001 [73] showed similar antitumor activity of disitimab vedotin in combination with pembrolizumab in 20 treatment-naïve patients with HER2-expressing (HER2-positive: IHC 3+, or IHC 2+ and ISH-positive or HER2-low: IHC 2+ and ISH-negative, or IHC 1+) advanced UC (confirmed ORR of 75%, with 35% achieving complete response). Neoadjuvant treatment with disitamab vedotin in combination with perioperative toripalimab [74] was associated with a pCR rate of 63.6% in a phase II trial including 47 patients with HER2-expressing (immunohistochemistry ≥ 1+ by local test) MIBC, with an even higher rate (84.6%) in a subgroup of HER2 3+ patients.

Data from phase III RC48-C016 [75] confirmed the efficacy of the first-line treatment of disitamab vedotin plus toripalimab compared to standard platinum-gemcitabine chemotherapy in 484 Chinese patients with advanced HER2-expressing (determined by the central laboratory to be IHC 1+, 2+ or 3+) UC: experimental arm was associated with longer PFS (median PFS 13.1 vs. 6.5 months, HR 0.36 95% CI 0.28–0.46 *p* < 0.0001) and OS (median OS 31.5 vs. 16.9 months, HR 0.54 95% CI 0.41–0.73 *p* < 0.0001) and higher ORR (76.1% vs. 50.2%), with more a favorable safety profile (incidence of grade 3 or higher TRAEs was 55.1% and 86.9%, respectively).

A similar phase III trial (SGNDV-001; ClinicalTrials.gov identifier NCT05911295) is currently exploring efficacy and safety of first-line disitamab vedotin plus pembrolizumab compared to standard chemotherapy in a worldwide population of HER2-expressing (IHC 1+ or greater) advanced UC.

Despite the discouraging results of the first studies with TKIs and monoclonal antibodies against HER2, the use of ADCs seems to provide greater activity and efficacy in HER2-selected UC; this could be related to the broader mechanism of action of ADCs, that acts by binding HER2 and leading to its internalization, exerting cytotoxic action of payload and potential bystander killing effect.

Based on recent data, HER2 targeting represents one of the most interesting future perspectives in the therapeutic landscape of UC; The FDA granted breakthrough therapy designation for disitamab vedotin monotherapy in second-line treatment of HER2-altered metastatic UC. In future, a combination of disitamab vedotin and ICI could be established as a first-line treatment in HER2-expressing UC patients.

## 7. Homologous Repair Recombination (HRR) Pathway Inhibition

The prevalence of mutations involving HRR genes (such as BRCA1 and BRCA2) is approximately 25–30% in urothelial bladder cancer [76,77], whereas slightly lower frequencies are observed in patients with UTUC. Use of poly ADP-ribose polymerase (PARP) inhibitors represents an effective therapeutic option in tumors harboring HRR gene alteration [78] through the simultaneous inhibition of two pathways involved in repairing both double- and single-strand DNA breaks (synthetic lethality). Preliminary data of the activity and efficacy of PARP inhibitors in patients with metastatic UC were derived from several clinical trials.

In phase II ATLAS [79], 97 patients with previously treated (with one or two prior lines of treatment) advanced UC received rucaparib regardless of HRR status. This trial did not meet its primary endpoint: there were no confirmed investigator-assessed objective responses in the overall population or in the subgroup of patients (*n* = 20) with HRR alterations. The disease control rate (DCR) was 11.6% and 15.8% in the overall population and in HRR-positive patients, respectively. Role of PARP inhibitors in maintenance setting was assessed in two phase II trials. In Meet-URO12 [80], 58 patients with molecularly unselected metastatic UC without evidence of progressive disease after 4–6 cycles of platinum-based first-line chemotherapy were randomly assigned in a 2:1 ratio to receive niraparib plus best supportive care (BSC) or BSC alone. The addition of maintenance therapy with niraparib did not show an improvement in PFS. Maintenance treatment with rucaparib significantly prolonged PFS in biomarker-selected UC patients [81] (defined as the presence of genomic loss of heterozygosity or somatic/germline mutation of DNA repair deficiency associated genes) compared to placebo in adaptive phase II platform ATLANTIS trial.

The presence of DNA damage may increase the formation of neoantigens, increasing sensitivity to immunotherapy. Based on this biological rationale, the activity of ICIs in combination with PARP inhibitors was explored. In the randomized, phase II BAYOU trial [82], durvalumab plus olaparib did not improve PFS compared to durvalumab alone in 154 platinum-ineligible patients with previously untreated metastatic UC. In 20% of patients with HRR gene mutation, combination treatment extended PFS compared to ICI alone (median PFS 5.6 vs. 1.8 months, HR 0.18 95% CI 0.06–0.47).

No PARP inhibitor has currently received therapeutic indications in UC and, although HRR targeting may represent an interesting therapeutic prospect, more substantial data are needed to be able to express a final judgment on this class of drugs.

Currently available data from selected prospective trials are reported in Table 1; a brief summary of the main features of selected ongoing trials is reported in Table 2.

**Figure 1:**
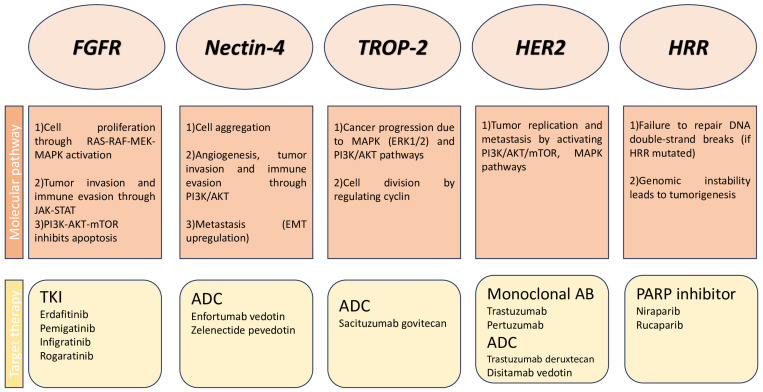
Schematic summary of the molecular pathway and related targeted therapies.

**Figure 2:**
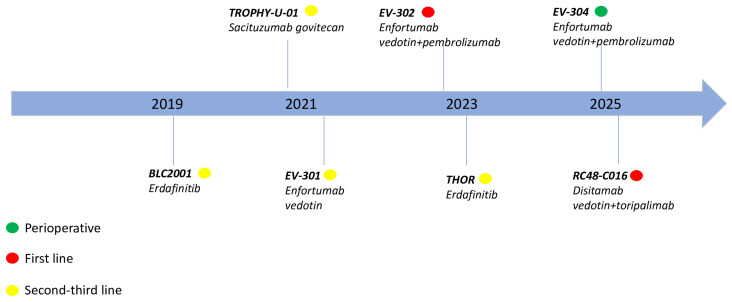
Evolution of the main targeted therapies in UC.

A schematic summary of the main molecular pathway and related targeted therapies in UC is reported in Figure 1; temporal evolution regarding the main pivotal trials of targeted therapies in UC is described in Figure 2.

## 8. Conclusions

Metastatic UC remains an incurable disease, characterized by poor prognosis. In the last few years, the recent advances in the genetic profiling of UC have sped up the idea of a precision medicine-based approach with the aim of selecting the most appropriate treatment dependent on the specific molecular alterations displayed by the single tumor. In addition, the advent of novel targeted therapies, such as checkpoint inhibitors and ADC, administered as monotherapy or in combination with commonly used drugs, could lead to a radical change in the therapeutic landscape, allowing a significant improvement in survival in the near future.

## Figures and Tables

**Table 1:** Summary of selected prospective trials regarding targeted agents in UC.

Molecular Pathway and Selection	Clinical Trial Identifier	Line/Setting	Phase	Treatment Arm(s)	Accrual	Primary Endpoint(s)	Results
FGFR inhibition (FGFR3/2 mutations or fusions)	NCT03390504 THOR (cohort 1, ICI-pretreated)	≥2	III	erdafitinib vs. docetaxel or vinflunine	266	OS	median OS 12.1 vs. 7.8 mos median PFS 5.7 vs. 2.7 mos grade ≥ 3 TRAEs 45.9% vs. 46.4%
FGFR inhibition (FGFR3/2 mutations or fusions)	NCT03390504 THOR (cohort 2, ICI-naive)	≥2	III	erdafitinib vs. pembrolizumab	351	OS	median OS 10.9 vs. 11.1 mos median PFS 4.4 vs. 2.7 mos grade ≥ 3 TRAEs 43.4% vs. 12.1%
FGFR inhibition (FGFR1/3 mRNA expression)	NCT03410693 FORT-1	≥2	II/III	rogaratinib vs. taxanes or vinflunine	175	OS	median OS 8.3 vs. 9.8 mos ORR 20.7 vs. 19.7% grade ≥ 3 TRAEs 43% vs. 39%
FGFR inhibition (FGFR3 mutations or fusions)	NCT02872714 FIGHT-201 (cohort A)	≥2	II	pemigatinib	204	ORR	ORR 17.8% and 23.3% (continuous and intermittent administration)
FGFR inhibition (FGFR3 mutations or fusions)	NCT03473743 Norse	1	II	erdafitinib + cetrelimab vs. erdafitinib	87	ORR and safety	ORR 54.5% vs. 44.2% 1-year OS rate 68% vs. 56% grade ≥ 3 TRAEs 45.5% vs. 46.5%
Nectin-4 targeting (unselected)	NCT03474107 EV-301	≥2	III	enfortumab vedotin vs. taxanes or vinflunine	608	OS	median OS 12.88 vs. 8.97 mos median PFS 5.55 vs. 3.71 mos grade ≥ 3 TRAEs 51.4% vs. 49.8%
Nectin-4 targeting (unselected)	NCT04223856 EV-302	1	III	enfortumab vedotin + pembrolizumab vs. carbo/cisplatin + gemcitabine	886	OS and PFS	median OS 33.8 vs. 15.9 mos median PFS 12.5 vs. 6.3 mos grade ≥ 3 TRAEs 57.3% vs. 69.5%
Nectin-4 targeting (unselected)	NCT03924895 EV-303	Perioperative	III	enfortumab vedotin + pembrolizumab vs. cystectomy alone (allocation to third arm with pembrolizumab monotherapy was stopped)	344	EFS	median EFS NR vs. 15.7 mos median OS NR vs. 41.7 mos pCR 57.1 vs. 8.6% grade ≥ 3 TRAEs 71.3% vs. 45.9%
Nectin-4 targeting (unselected)	NCT03288545 EV-103 (cohort H)	Neoadjuvant	Ib/II	enfortumab vedotin	22	pCR rate	pCR 36.4%
Nectin-4 targeting (unselected)	NCT04561362 Duravelo-1 (cohort B7)	1	I–II	zelenectide pevedotin + pembrolizumab	22	ORR	ORR 65% grade ≥ 3 TRAEs 68.2%
TROP-2 targeting (unselected)	NCT04527991 TROPiCS-04	≥2	III	sacituzumab govitecan vs. taxanes or vinflunine	711	OS	median OS 10.3 vs. 9.0 mos median PFS 4.2 vs. 3.6 mos grade ≥ 3 TRAEs 67% vs. 35%
TROP-2 targeting (unselected)	NCT03547973 TROPHY-U-01 (cohort 3)	≥2	II	sacituzumab govitecan + pembrolizumab	41	ORR	ORR 41% median PFS 5.2 mos median OS 12.7 mos grade ≥ 3 TRAEs 61%
TROP-2 targeting (unselected)	NCT05226117 SURE-01	Neoadjuvant	II	sacituzumab govitecan	33	pCR rate	pCR 36.4%
HER2 targeting (IHC ≥ 1+)	NCT05302284 RC48-C016	1	III	disitamab vedotin + toripalimab vs. carbo/cisplatin + gemcitabine	484	OS and PFS	median OS 31.5 vs. 16.9 mos median PFS 13.1 vs. 6.5 mos grade ≥ 3 TRAEs 55.1% vs. 86.9%
HER2 targeting (IHC 3+ or 2+)	NCT03507166 RC48-C005	≥2	II	disitamab vedotin	43	ORR	ORR 51.2% median PFS 6.9 mos median OS 13.9 mos grade ≥ 3 TRAEs 58%
HER2 targeting (IHC 3+ or 2+)	NCT04482309 DESTINY-PanTumor02	≥2	II	trastuzumab deruxtecan	41	ORR	ORR 39% Median PFS 7.0 mos grade ≥ 3 TRAEs 41.5%
HER2 targeting (IHC ≥ 1+)	NCT04879329 RC48G001 (cohort C)	1	II	disitamab vedotin + pembrolizumab	20	ORR	ORR 75% grade ≥ 3 TRAEs 45%
HRR targeting (unselected)	NCT03945084 Meet-URO12	Maintenance	II	niraparib vs. best supportive care	58	PFS	median PFS 2.1 vs. 2.4 mos
HRR targeting (DNA repair deficiency)	2015-003249-25 ATLANTIS	Maintenance	II	rucaparib vs. placebo	40	PFS	median PFS 35.3 vs. 15.1 weeks

Abbreviations: NCT: number of clinical trials (https://clinicaltrials.gov/); TRAEs: Treatment-Related Adverse Events; mos: months; NR: not reached.

**Table 2:** Selected ongoing trials regarding targeted agents in UC.

Molecular Pathway and Selection	Clinical Trial Identifier	Line/Setting	Phase	Treatment Arm(s)	Primary Endpoint(s)	Recruitment Status	Target Accrual
FGFR inhibition (FGFR3/2 mutations or fusions)	2022-002586-15 SOSUG-NEOWIN	Neoadjuvant	II	erdafitinib, erdafitinib + cetrelimab	pCR and tumor downstaging rate	Recruiting	90
FGFR inhibition (FGFR3/2 mutations or fusions)	NCT04963153 ETCTN 10483	≥2	Ib	erdafitinib + enfortumab vedotin	Safety and RP2D	Recruiting	30
Nectin-4 targeting (unselected)	NCT04700124 EV-304	Perioperative	III	enfortumab vedotin + pembrolizumab vs. cisplatin + gemcitabine	pCR and EFS	Active, not recruiting	780
Nectin-4 targeting (unselected)	NCT06225596 Duravelo-2	1 (cohort 1) ≥2 (cohort 2)	II–III	cohort 1: zelenectide pevedotin + pembrolizumab vs. carbo/cisplatin + gemcitabine (eventually followed by avelumab) cohort 2: zelenectide pevedotin	PFS (cohort 1) ORR (cohort 2)	Recruiting	641 (cohort 1) 315 (cohort 2)
TROP-2 targeting (unselected)	NCT05226117 SURE-01	Noeadjuvant	II	sacituzumab govitecan	pCR	Recruiting	44
TROP-2 targeting (unselected)	NCT05535218 SURE-02	Perioperative	II	sacituzumab govitecan + pembrolizumab	clinical CR	Active, not recruiting	48
HER2 targeting (IHC ≥ 1+)	NCT05911295 SGNDV-001	1	III	disitamab vedotin + pembrolizumab vs. carbo/cisplatin + gemcitabine	PFS and OS	Recruiting	400

Abbreviations: NCT: number of clinical trial (https://clinicaltrials.gov/).

## Data Availability

No new data were created or analyzed in this study. Data sharing is not applicable to this article.
